# Orthostatic blood pressure changes among adults in Ekiti state, Nigeria: Impact of socio‐demographic, anthropometric, and biophysical factors

**DOI:** 10.14814/phy2.70792

**Published:** 2026-02-25

**Authors:** Oyesanmi A. Fabunmi, Mayowa J. Adeniyi, Andrea Lenting, Thulani Masilela, Dina C. (Christa) Janse van Rensburg

**Affiliations:** ^1^ Section Sports Medicine, Faculty of Health Sciences University of Pretoria Hatfield South Africa; ^2^ Health‐Awareness, Exercise and Cardio‐Immunologic Research Unit (HECIRU), Department of Physiology, College of Medicine Ekiti State University Ado‐Ekiti Ekiti State Nigeria; ^3^ Sport Exercise Medicine and Lifestyle Institute (SEMLI), Faculty of Health Sciences University of Pretoria Hatfield South Africa; ^4^ Departments of Physiology Federal University of Health Sciences Otukpo Benue Nigeria; ^5^ Departments of Physiology, School of Medicine and Pharmacy University of Rwanda Kigali Rwanda

**Keywords:** adults, Ekiti state, hemodynamic changes, Nigeria, orthostasis

## Abstract

Orthostatic intolerance is the inability to maintain upright posture, a key manifestation of autonomic dysfunction that can lead to orthostatic hypotension (OH). We aimed to investigate orthostatic blood pressure (BP) changes among cohorts living in Ekiti State and to explore plausible underlying factors. We encompassed 140 participants (28% male; 72% female; age range: 18–70 years) in our preliminary study. Socio‐demographic and anthropometric indices were collected using appropriate tools. Orthostatic responses were assessed at baseline, standing, and sitting following standardized protocols. Systolic (BP) decreased significantly by 4% after 1‐min standing (130 ± 22 mmHg) compared to baseline (137 ± 20 mmHg). Diastolic (BP) increased significantly by 6.1% at 3 min (87 ± 13 mmHg) and 4.8% at 5 min (86 ± 12 mmHg) compared to baseline (82 ± 11 mmHg) (*p* < 0.001). Occupation (traders) and education level (secondary) are positively associated with mean arterial pressure (MAP) at 1‐ and 3‐min standing times (*p* < 0.05). Orthostatic MAP at 1, 3, and 5 min correlated negatively with height but positively with body mass index (BMI), hip, and waist circumference (*p* < 0.05). These preliminary findings suggest a potential role for body composition and lifestyle in regulating autonomic cardiovascular function in response to postural changes.

## 1 INTRODUCTION

Orthostatic intolerance (OI) is the inability to tolerate upright posture, which is a key manifestation of autonomic dysfunction that can lead to orthostatic hypotension (OH) (Lambert & Lambert, 2014). OH is a common cardiovascular disorder that is usually overlooked, with or without signs of underlying neurodegenerative disease (Freeman et al., 2011). It is characterized by a persistent decline in systolic/diastolic blood pressure of at least 20/10 mm Hg upon standing or during a head‐up tilt (HUT) test of at least 60° (Guaraldi & Calandra‐Buonaura, 2023). It is associated with debilitating symptoms, falls, syncope, cognitive impairment, and risk of death (Fedorowski et al., 2022). Certain factors relating to age and gender are known to influence OH (Zambach et al., 2023). Previous evidence suggests that about 30% of individuals older than 70 years and 5%–10% of middle‐aged individuals experience OH (Ricci, De Caterina, & Fedorowski 2015; Shin et al., 2004; Zambach et al., 2023). The occurrence of OH in older adults represents a potential intrinsic risk factor for falls in these individuals (Shaw, Loughin, Robinovitch, & Claydon, 2015). Evidence also suggests that an early‐onset OH increases risks of myocardial infarction (MI), stroke, and dementia in young adults, underscoring the necessity to pay additional attention to the cardiovascular health and neurocognitive status among susceptible individuals (Liang et al., 2024).

Age‐related susceptibility to OH is orchestrated by impaired heart rate response, vascular changes (arterial stiffening and venous pooling due to changes in collagen/elastin ratio), and reduced baroreflex sensitivity (Saz‐Lara et al., 2023). These changes underscore the potential role of OH and increased risk of major cardiovascular events (MACEs) and mortality in high‐risk (Ricci, Fedorowski, et al., 2015). Local stiffening of the regions in central arteries, where high‐pressure baroreceptors are located, can make the arterial wall less responsive to changes in luminal pressure, thereby diminishing the mechanical sensitivity and effectiveness of the baroreceptor reflex arc (Srivastava et al., 2023). Furthermore, the role of gender‐related disparity on orthostatic changes suggests that females are more likely to experience OH when compared with their age‐matched males, which is attributed to the low muscle sympathetic nervous activity, a critical component of baroreflex sensitivity (Adeniyi et al., 2022; Hart et al., 2009). The neurovascular regulatory mechanism is relatively less capable of compensating for changes in blood pressure (BP) and blood volume due to low renin levels in females (Medina et al., 2020; Raj et al., 2005). This implication may compromise the functionality of the renin‐angiotensin‐aldosterone system in regulating blood pressure, contributing to a higher risk of OH (Raj et al., 2005).

Orthostatic BP variability denotes short‐term dynamic fluctuations in BP after postural transition to standing and the early period of upright posture (Ní Bhuachalla et al., 2015). It is distinct from sustained orthostatic hypotension as it requires continuous BP monitoring during the first 60–180 s in the upright position (Ní Bhuachalla et al., 2015, 2019). It is also a reflection of autonomic abnormality and is independently associated with visual impairment, cognitive decline, falls, and cardiovascular risk among high‐risk patients (Ní Bhuachalla et al., 2015, 2019; van Wijnen et al., 2017). Emerging evidence has highlighted the implications of climacteric conditions and alterations in BP variability among high‐risk individuals (Narita et al., 2021). Evidence suggests that the risk of OH is higher during the summer due to heat stress (Schlader et al., 2015). Increased heat stress can increase the risk of dehydration due to excessive fluid loss and reduced blood volume, among other factors. All this contributes to a decline in orthostatic BP observed in a hot environment (Han et al., 2019; Ferreira et al., 2024). In the context of Nigerian demography, plausible contributory factors underlying variations in BP and the prevalence of OH remain underexplored (Adeniyi et al., 2024). Emerging evidence suggests an association of socio‐demographic and lifestyle factors with high prevalence of CVD, such as hypertension, in Nigeria (Adeke et al., 2024). As the burden of CVD in low and middle‐income countries (LMICs) is projected to increase over the years, adopting a cost‐effective screening strategy for CVD prevention and control in various population settings is essential for enhancing health and clinical outcomes, particularly in low‐resource settings at the national and subnational levels (Wurie & Cappuccio, 2012). Thus, the aim of this study is to assess orthostatic BP changes among residents living in the Ekiti State University community and to explore the plausible underlying factors.

## 2 METHODOLOGY

### 2.1 Study design, setting, and population

This cross‐sectional observational study involved 140 participants and was conducted in the laboratory unit of the Department of Physiology at Ekiti State University, Ado‐Ekiti, Ekiti State, Nigeria, from December 2024 to March 2025. The site was chosen because no similar study has been previously executed in this community. A snowball sampling technique was adopted during the course of the study. Considerations were given only to participants aged 18 years or older, and informed written consent was obtained from all participants before the commencement of the study and data collection. Participants were shown a simulation demonstration to sensitize them about the concept and approach of the study (Rupnik et al., 2002). Furthermore, participants were thoroughly interviewed via a semi‐structured questionnaire to obtain their medical history for screening and eligibility to participate in the study. All the procedures were conducted in quiet and comfortable rooms to minimize the influence of social facilitation on the measured parameters. This study was approved by the Ethics Committee of Ekiti State University Teaching Hospital (No: EKSUTH/A67/2024/12/008). The study adhered strictly to the principles outlined in the Declaration of Helsinki concerning human participants (Lancet T, 2000).

### 2.2 Experimental protocol

#### 2.2.1 Determination of socio‐demographic and daily physical activity (DPA) status

Data collection sheet on socio‐demographics and DPA status was adapted from the WHO STEPS Instrument for Non‐Communicable Disease Risk Factor Surveillance (World Health Organization, 2021) and the International Physical Activity Questionnaire (IPAQ), which was developed as an instrument for cross‐national monitoring of physical activity and inactivity in diverse settings (Craig et al., 2003).

#### 2.2.2 Measurement of anthropometric, metabolic and respiratory indices

Prior to taking anthropometric measurements, participants were instructed to remove their shoes, coats, and hats. Body weight (kg) was then measured using a digital scale, with the value recorded once the reading stabilized. Height (m) was measured with a stadiometer. Body Mass Index (BMI) was calculated as body weight (kg) divided by the square of height (m). Waist, neck, and hip circumferences were measured using a tape measure following a standardized procedure by a trained technician as previously described (Polymeris et al., 2014). Blood glucose levels were determined from finger‐prick blood samples using a glucometer (Accu‐Check, Roche, Biochemical Laboratory, Germany) as previously described (Pickering & Marsden, 2014). Core body temperature (CBT) was measured using the non‐contact (Infrared) digital thermometer (Casey Kandio Technology, China). Peripheral oxygen saturation was measured using a pulse oximeter (Beurer Pulse Oximeter PO, Germany).

#### 2.2.3 Orthostatic intolerance test

**FIGURE 1:**
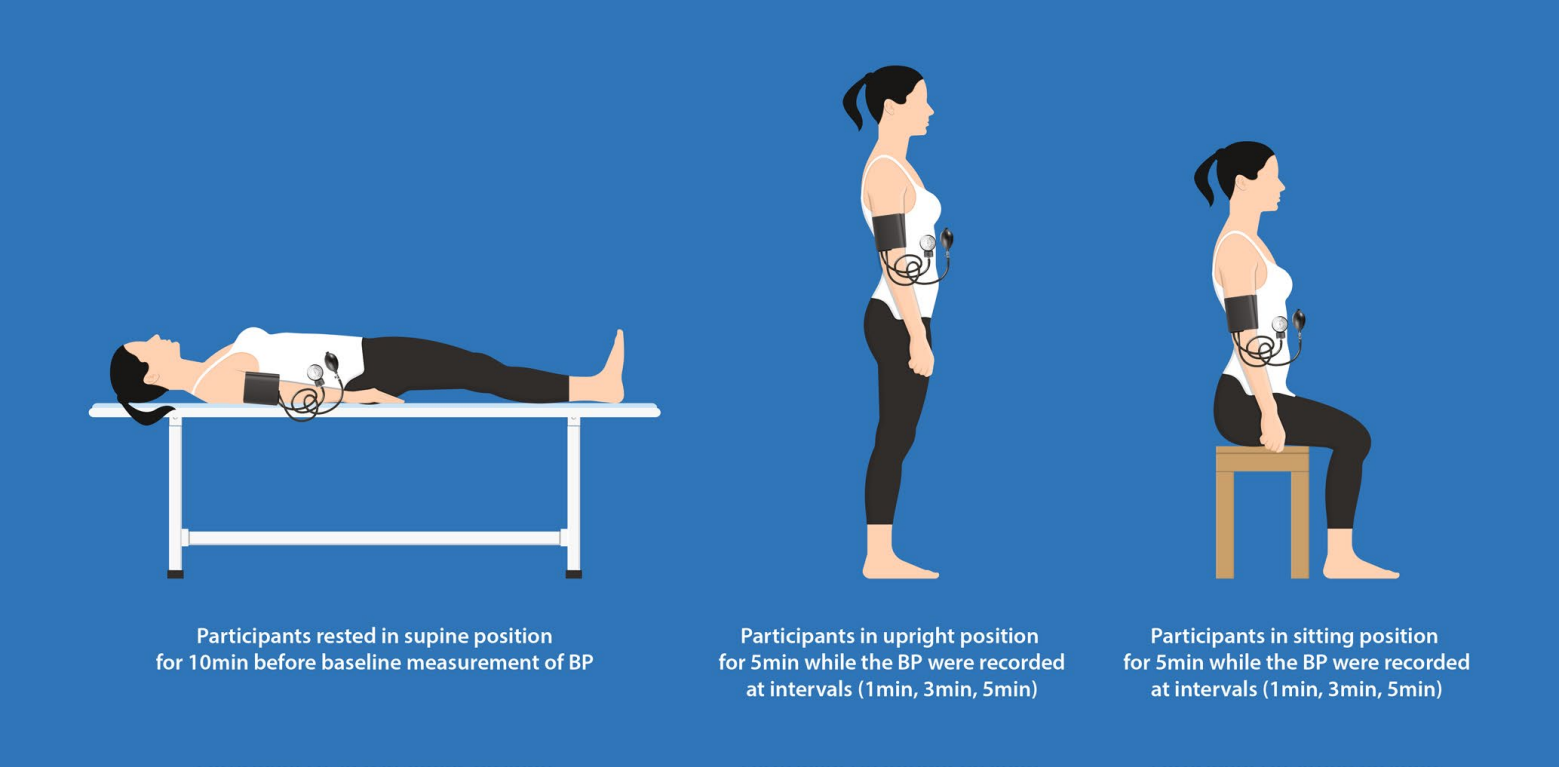
The schematic overview of the assessment of orthostasis at various intervals during baseline, upright and sitting positions. Upright posture caused a small SBP drop and DBP rise from supine, while sitting showed no change. Occupation, education, and body measures were linked to MAP changes in upright posture.

Orthostatic responses were conducted via active standing and sitting tests (Figure 1). The study was done in the quiet Physiology Laboratory at an ambient temperature of 25°C. Before the commencement of any measurement, all instruments were well‐calibrated to minimize error and imprecision. The participants were familiarized with the experimental procedure, including the performance of orthostasis and how to report feelings of exertion and dizziness through a simulation exercise. During the study, participants were relaxed in a supine position for 10 min to maintain a stable fluidic hemodynamic state before taking baseline hemodynamic parameters. Subsequently, participants assumed an active standing position, and hemodynamic parameters were measured and recorded at 1, 3, and 5 min, respectively, after they had been in the supine position for a specified time (Shin et al., 2004). Thereafter, participants proceeded to a sitting position in a chair with their hands hanging down naturally, knees bent at right angles, feet on the floor, and back without any support, while hemodynamic parameters were measured and recorded at 1, 3, and 5 min of sitting, respectively (Tao et al., 2020).

#### 2.2.4 Measurement of hemodynamic parameters

The hemodynamic parameters, such as systolic blood pressure (SBP), diastolic blood pressure (DBP), and mean arterial pressure (MAP), were assessed via the automated Omron BP7000 Upper Arm Sphygmomanometer (Iris Global Care, China). The cuff of the sphygmomanometer was tied an inch above the elbow to measure the blood pressure changes by a trained technician. The mean arterial blood pressure was calculated as diastolic blood pressure + 1/3 of pulse pressure (DeMers & Wachs, 2019). BP was measured twice in each position (standing and sitting), and the average of the two measurements was recorded. For systolic pressure, a mild drop was defined as 1–9 mmHg, intermediate as 10–19 mmHg and OH as 20 mmHg or more. The corresponding definitions for diastolic pressure were 1–4, 5–9, and 10 mmHg or more (Weiss et al., 2004).

#### 2.2.5 Statistical analysis

Analyses were performed using SPSS. Firstly, normality testing was done to determine the distribution of data before analysis. A statistical test was performed using ANOVA for comparisons across repeated measures, where appropriate (supine, 1, 3, and 5 min of postural changes). Correlation analysis was performed using the Chi‐square test and Pearson's correlation, where appropriate. Categorical data were expressed as a frequency and percentage, while measurement data (continuous outcomes) were expressed as the mean ± standard deviation (SD) with the respective effect size (ES). A value of *p* < 0.05 was taken as statistical significance.

## 3 RESULTS

### 3.1 Determination of socio‐demographic characteristics, DPA status, anthropometric, metabolic, and respiratory indices

Tables 1, 2, 3 shows the socio‐demographic characteristics, DPA status, anthropometric, metabolic, and respiratory indices of the participants. In Table 1, females outnumbered males. In terms of age, the majority were young adults between 18 and 27 years, compared with other age groups, including older adults and middle‐aged adults. Most of the participants fall under the tertiary education level, while the primary education level had the lowest number. In terms of occupation, most of the participants were students, followed by traders, while administrators were the least represented. In terms of religion, most participants identify as Christians, compared to Muslims. In terms of DPA, the majority of participants engaged in moderate‐intensity DPA, and walking was the preferred type of activity compared to other types, such as jogging and running (Table 2). The average BMI, blood glucose, CBT and SP0_2_ of the participants were within the normal range, respectively (Table 3).

**TABLE 1:** Socio‐demographic characteristics of the participants.

Parameters		Category	Frequency (*n*)	Percentage (%)
Socio‐demographics indices	Sex	Male	41	30
		Female	99	70
	Age (years)	18–27	58	41
		28–37	2	1
		38–47	4	3
		48–57	14	10
		58–67	20	15
		>68	42	30
	Education	Primary	14	10
		Secondary	43	30
		Tertiary	83	60
	Occupation	Administrator	1	0.7
		Artisan	6	4
		Driver	2	1.4
		Farmer	8	6
		Student	54	38.3
		Teacher	26	18.6
		Trader	43	31
	Religion	Christian	137	98
		Muslim	3	2

**TABLE 2:** DPA status of the participants.

Parameters		Category	Frequency (*n*)	Percentage (%)
Types, duration and weekly daily physical activity (DPA) level	Walking	Min/Day		
		1–4	5	3.6
		5–10	12	7.1
		>10	123	89.3
		Days/Week		
		1–2	5	3.6
		3–4	12	7.1
		>4	123	89.3
	Jogging	Min/Day		
		None	93	66.4
		5–10	13	9.3
		>10	34	24.3
		Days/Week		
		None	93	66.4
		3–4	13	9.3
		>4	34	24.3
	Running	Min/Day		
		None	129	92
		1–4	7	5
		5–10	4	3
		Days/Week		
		None	129	92
		>4	11	8
	DPA level/week	Intensity		
		Low	4	2.9%
		Moderate	136	97.1%

**TABLE 3:** Anthropometric, metabolic, and respiratory indices of the participants.

Anthropometric indices	Mean ± SD
Body weight (kg)	59.1 ± 0.98
Height (m)	1.62 ± 0.83
Body mass index (BMI; kg/m^2^)	22.5 ± 0.4
Hip circumference (cm)	97.4 ± 1.2
Waist circumference (cm)	89.6 ± 1.2
Wasit‐hip ratio	0.92 ± 0.01
Neck circumference (cm)	35.6 ± 0.27
Metabolic and respiratory indices	
Glucose level (mmol/l)	5.7 ± 0.2
CBT (°C)	36.9 ± 0.1
SPO_2_ (%)	98.2 ± 0.14

### 3.2 Effect of orthostatic duration on hemodynamic parameters

**FIGURE 2:**
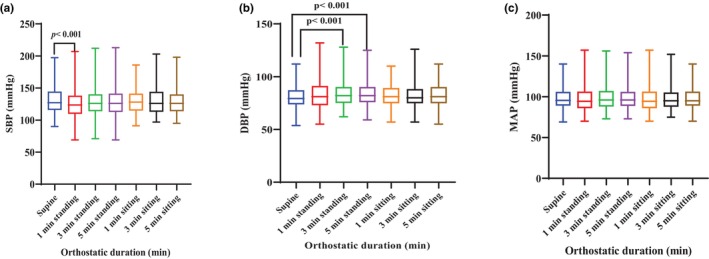
Effect of orthostatic duration on participant hemodynamic parameters (a) SBP, (b) DBP, (c) MAP. *Significant difference from supine at *p* < 0.05, Effect size SBP: 0.2, DBP: −0.2.

The effect of orthostatic duration on SBP showed that standing for 1 min caused a significant reduction in systolic blood pressure when compared to the supine position (*p* < 0.001) (Figure 2a). Furthermore, standing for 3 and 5 min resulted in a significant increase in DBP compared to the supine position, respectively (*p* < 0.001) (Figure 2b). However, orthostatic durations showed no significant effect on MAP when compared with the supine position (*p* > 0.05) (Figure 2c).

### 3.3 Relationship between orthostatic MAP and socio‐demographic indices

Occupation was positively associated with mean arterial blood pressure following 1 min (*p* = 0.004) and 3 min of standing (*p* = 0.034) (Table 4). In the occupation, participants who are traders exhibited moderate change in 1‐min and 3‐min orthostatic MAP (Table S4A, supplementary file). Furthermore, the educational level was positively associated with 1‐min (*p* = 0.001) and 3‐min orthostatic MAP (*p* = 0.016) (Table 4). The participants with secondary level educational status had moderate change in 1‐min and 3‐min orthostatic MAP (Table S4B, supplementary file).

**TABLE 4:** Relationship between orthostatic mean arterial pressure and socio‐demographic indices of the participants.

Orthostatic MAP	Socio‐demographic indices				
Chi square		Occupation	Age	Gender	Educational level
1 min standing	Low	37.996 (*p* = 0.004)*	8.179 (*p* = 0.225)	26.580 (*p* = 0.087)	29.093 (*p* = 0.001)*
	Moderate				
	High				
3 min standing	Low	30.408 (*p* = 0.034)*	5.886 (*p* = 0.436)	23.444 (*p* = 0.174)	20.242 (*p* = 0.016)*
	Moderate				
	High				
5 min standing	Low	26.936 (*p* = 0.08)	6.225 (0.398)	20.430 (*p* = 0.309)	16.505 (*p* = 0.057)
	Moderate				
	High				

* *p* < 0.05.

### 3.4 Relationship between orthostatic MAP and anthropometric indices

As shown in Table 5, 1‐, 3‐, and 5‐min orthostatic MAP correlated negatively with height (*p* < 0.001). Furthermore, 1‐, 3‐, and 5‐min orthostatic MAP correlated positively with BMI (*p* < 0.001). Similarly, 3‐ and 5‐min orthostatic MAP showed a positive correlation with waist circumference (*p* < 0.038). Lastly, 1, 3, and 5 min orthostatic MAP correlated positively with hip circumference (*p* < 0.05).

**TABLE 5:** Relationship between orthostatic mean arterial pressure and anthropometric indices of the participants.

Orthostatic MAP	Anthropometric indices						
Pearson correlation (r)	Weight	Height	BMI	Waist circumference	Hip circumference	Neck circumference	Waist/Hip ratio
1 min standing	0.143 (*p* = 0.135)	−0.34 (*p* < 0.001)*	0.389 (*p* < 0.001)*	0.179 (*p* = 0.06)	0.271 (*p* = 0.004)*	0.121 (*p* = 0.204)	−0.113 (*p* = 0.24)
3 min standing	0.13 (*p* = 0.17)	−0.405 (*p* < 0.001)*	0.416 (*p* < 0.001)*	0.197 (*p* = 0.038)*	0.282 (*p* = 0.003)*	0.119 (*p* = 0.213)	−0.1 (*p* = 0.296)
5 min standing	0.144 (*p* = 0.132)	−0.407 (*p* < 0.001)*	0.434 (*p* < 0.001)*	0.197 (*p* = 0.038)*	0.299 (*p* < 0.001)*	0.136 (*p* = 0.156)	−0.127 (*p* = 186)

* *p* < 0.05.

### 3.5 Relationship between orthostatic MAP, blood glucose, CBT, and oxygen saturation index

As shown in Table 6, 1‐, 3‐, and 5‐min orthostatic MAP showed no significant association with the participants' blood glucose, CBT, and SPO_2_, respectively (*p* > 0.05).

**TABLE 6:** Relationship between orthostatic MAP, metabolic, and respiratory indices of the participants.

Orthostatic MAP	Metabolic and respiratory indices		
Pearson correlation (r)	Blood glucose	SPO_2_	CBT
1 min standing	0.0307 (*p* = 0.701)	0.008 (*p* = 0.932)	0.091 (*p* = 0.34)
3 min standing	0.017 (*p* = 0.856)	0.003 (*p* = 0.974)	0.095 (*p* = 0.32)
5 min standing	−0.007 (*p* = 0.944)	−0.031 (*p* = 0.75)	0.103 (*p* = 0.281)

*Note*: Significant difference, *p* < 0.05.

## 4 DISCUSSION

This study assessed orthostatic BP changes among cohorts living in Ekiti State, Nigeria and also explored the possible underlying contributing factors. The main findings of our preliminary study showed a slight, significant drop in the SBP and an increase in the DBP during upright posture from baseline (supine), while the hemodynamic changes during sitting remained unchanged across the time points. The potential role of sociodemographic and biophysical parameters in blood pressure regulation showed that occupation, education level, and anthropometric measures were significantly associated with changes in MAP during upright posture. The observed alterations in hemodynamics highlight the potential role of OH as an emerging risk factor for MACEs (Juraschek et al., 2023; Liang et al., 2024).

In our study, we observed a significant alteration in hemodynamic measurements at various time points during the standing phase (Figure 2). There was a slight decrease in SBP by 4% after 1‐min standing (130 ± 22 mmHg) from baseline (137 ± 20 mmHg) (*p* < 0.001). DBP increased slightly by 6.1% at 3 min (87 ± 13 mmHg) and 4.8% at 5 min (86 ± 12 mmHg) from baseline (82 ± 11 mmHg) (*p* < 0.001). It is noteworthy that our findings did not match the threshold to ascertain OH (Weiss et al., 2004). However, they corroborate a previous population‐based longitudinal study that reported a slight drop in SBP upon standing for 2 min from a supine position in the morning (Weiss et al., 2004), and previous findings where DBP increased during supine‐to‐standing testing among middle‐aged healthy participants (Shaw et al., 2017). Notably, there were no changes in MAP during the postural transition across the time points (*p* > 0.05). More so, the hemodynamic measurements in the participants remained unaltered across the time points while sitting (*p* > 0.05), which is in trend with a previous study that reported no changes in hemodynamic parameters during a passive seated orthostatic stress test among healthy volunteers (Shaw, Loughin, Mackey, et al., 2015). The stabilization of BP during postural changes indicates a form of physiological resilience, which is a characteristic of an efficient vascular and cardiac system in healthy individuals.

It is well known that the impact of climate change and extreme weather conditions, such as heat stress and those associated with Harmattan weather, can lead to deleterious health outcomes in high‐risk individuals (Abdul‐Nabi et al., 2025; Adeniyi et al., 2024). For instance, the Harmattan season in Nigeria occurs between December and March and is characterized by dusty, cold winds and haze (Oladele, 2007). The undesirable effect of the dusty, cold, hazy wind from the Harmattan season is linked to alterations in hemodynamic parameters (BP), respiratory disorder, and an increased rate of hospitalization among susceptible individuals (Adeniyi et al., 2024; Bauer et al., 2022; Brook et al., 2023; Okeahialam & Zoakah, 2005). The resultant increase in BP occurs due to the disruption of the endocardium ecosystem and the tunica intima of blood vessels, leading to impairment in blood flow patterns, which causes turbulence and increases the likelihood of tissue damage, as well as several other cardiovascular complications (Jiménez et al., 2010).

More so, the increased risk of OH due to the impact of heat stress is driven by several factors that involve multiple physiological systems, including those associated with the arterial and venous circulations (Kollias et al., 2020; Schlader et al., 2015). The deleterious changes in vascular resistance, cardiac output, and blood distribution contribute to the inability to maintain cerebral perfusion during heat and orthostatic stress (Schlader et al., 2015). Evidence suggests that numerous compensatory mechanisms work integratively to maintain BP immediately after a postural transition from supine to upright (Juraschek et al., 2018; Ricci, De Caterina, & Fedorowski 2015). In principle, gravitationally induced redistribution of intravascular volume toward the lower extremities is sensed by high‐pressure arterial baroreceptors located in the carotid sinus and aortic arch, as well as low‐pressure cardiopulmonary baroreceptors primarily situated in the right atrium (Ricci, De Caterina, & Fedorowski 2015). The carotid baroreceptor reflex is pivotal in this process, mediating an increase in sympathetic outflow and suppression of parasympathetic tone, which collectively promote catecholamine release, peripheral vasoconstriction, and elevated heart rate (Jones et al., 2012). Thus, abnormalities in any of these processes can destabilize the BP while standing, which can lead to OH during postural transition among susceptible individuals with an increased risk of CVD (Jones et al., 2012).

Emerging evidence has highlighted the potential role of several sociodemographic and biophysical factors and the risk of CVD, particularly in lower and middle‐income countries (Martens et al., 2023; Sun et al., 2015). In our study, we demonstrated an association between orthostatic BP and several socio‐demographic and biophysical factors. In terms of socio‐demographic factors, trade occupation and secondary education levels were positively associated with orthostatic MAP at 1‐ and 3‐min standing times in the participants (*p* < 0.05). This finding underscores the importance of trading as the most prevalent occupation and secondary education as the most prevalent educational level in predicting BP changes among participants. Our evidence also aligns with the trend of previous studies that highlight an association between low levels of education and an increased risk of cardiovascular events extending through middle and older adulthood (Garfein et al., 2025; Siren et al., 2014). Our findings also corroborate previous studies that reported an increased risk of cardiovascular events among traders in Western and Northern Nigeria (Achonu et al., 2022; Daniel et al., 2023) as well as workers from other regions in West Africa (Bosu, 2016). Taken together, our findings further strengthen the potential role of occupation and education level in predicting orthostatic BP changes as an emerging risk factor for CVD among vulnerable individuals.

In terms of anthropometrics, orthostatic MAP at 1, 3, and 5 min correlated negatively with height but positively with BMI, hip circumference, and waist circumference (*p* < 0.05), which is also in tandem with previous studies on both adult and young populations (Ejheisheh et al., 2022; Katamba et al., 2021; Staub et al., 2018). Although age, sex and daily physical activity level are not associated with orthostatic MAP in our study. However, this does not preclude the role of these variables and the risk of MACEs, as indicated in other previous studies (Ji et al., 2024; Stamatakis et al., 2025). For instance, vigorous intermittent lifestyle physical activity (VILPA) in various forms, ranging from walking to jogging and running, is a known countermeasure to mitigate the risk of CVD among susceptible individuals (Goswami et al., 2017; Stamatakis et al., 2025). Interestingly, our study revealed that most participants were engaged in moderate‐intensity DPA (Table 2), which may have contributed to the observed stabilization of BP during postural changes among the participants. Put together, our findings underscore the importance of educational awareness and personalized lifestyle modifications in reducing the risk of CVDs among susceptible individuals.

The strength of our study lies in the inclusion of a simulation exercise to sensitize the participants about the testing procedure. Growing evidence suggests that simulated practice learning is crucial in healthcare education, a view also supported by professionals and policymakers in certain regions of the world (Harrison et al., 2024; Kononowicz et al., 2019; Rayamajhi et al., 2024). Additionally, staff received rigorous training to follow standardized OH protocols, along with other relevant factors to minimize imprecision and bias. Moreover, measurements were performed in duplicates, and the average values were calculated to minimize errors, inconsistencies and imprecision. The main limitations of our study include a small sample size and the fact that our assessment was done in a community‐based ambulatory population during the Harmattan season and not at any other time during the year, which poses a significant limitation to unravel the role of seasonal changes and BP variability. Furthermore, other biochemical indicators of MACEs were not measured, and there was limited mechanistic data to unravel the implication of BP changes among the cohorts, as our preliminary results are primarily observational. Thus, further longitudinal studies are needed, and our findings should be replicated in other cohorts from different geographical regions, while considering the role of seasonal changes on BP variability and related outcomes.

Notably, the outcome of our study highlights the clinical significance of assessing orthostatic BP changes, which is crucial for refining cardiovascular risk stratification. It also gives insight into the distinction between traditional orthostatic hypotension, which is sustained, and orthostatic blood pressure variability, which requires continuous BP monitoring during postural changes for accurate detection that may not meet traditional hypotension criteria. Thus, the clinical implications of our study extend to the importance of effective assessment of OH, which can serve a crucial practical purpose in the clinical assessment and management of CVDs among high‐risk individuals. By making the diagnostic criteria for OH easier to assess, there is an opportunity to improve care for individuals with varied health challenges in low‐resource settings within LMICs, regardless of whether climate change is present.

## 5 CONCLUSION

The changes in orthostatic BP observed among cohorts residing in Ekiti State during the harmattan season were associated with occupation, educational level, and anthropometric indices. These preliminary findings suggest that body composition and lifestyle factors may impact autonomic cardiovascular regulation in response to postural changes. Thus, increased awareness about orthostatic BP changes should be taken into account in the clinical management of MACEs among high‐risk individuals.

## AUTHOR CONTRIBUTIONS

Oyesanmi A. Fabunmi and (Christa) Janse van Rensburg developed the study concept. Oyesanmi A. Fabunmi and Mayowa J. Adeniyi orchestrated the overall planning and content of the manuscript. Oyesanmi A. Fabunmi composed and wrote the main manuscript (first and final draft). Andrea Lenting helped review the manuscript for clarity and brevity. All authors reviewed and approved the final manuscript for submission.

## FUNDING INFORMATION

The authors have nothing to report.

## CONFLICT OF INTEREST STATEMENT

The authors declare no conflict of interest.

## Supporting information


**Table S2.** (A) Association between orthostatic MAP and occupation. (B) Association between orthostatic MAP and educational status.

**Table S4.** (A) Association between orthostatic MAP and occupation. (B) Association between orthostatic MAP and educational status.

## Data Availability

Data generated or analyzed during this study are available from the corresponding author upon reasonable request.
