# Supplementary material for: Orthostatic blood pressure changes among adults in Ekiti state, Nigeria: Impact of socio‐demographic, anthropometric, and biophysical factors
Source: Physiol Rep. 2026 Feb 25;14(4):e70792. doi: 10.14814/phy2.70792 (PMC12935748; doi:10.14814/phy2.70792)
Supplement: Supplementary file 1 — Table S2. (A) Association between orthostatic MAP and occupation. (B) Association between orthostatic MAP and educational status. [file PHY2-14-e70792-s002.doc]

*Table 2A.* *Association between orthostatic MAP and occupation*

| Orthostatic MAP | Occupation | | | | | | | |
| --- | --- | --- | --- | --- | --- | --- | --- | --- |
| Count | | Administration | Artisan | Driver | Farmer | Student | Teacher | Trader |
| 1 min standing | Low | 0 | 1 | 1 | 1 | 17 | 10 | 6 |
|  | Moderate | 2 | 5 | 0 | 8 | 7 | 15 | 28 |
|  | High | 0 | 0 | 0 | 0 | 0 | 1 | 8 |
| 3 min standing | Low | 0 | 2 | 1 | 1 | 13 | 8 | 3 |
|  | Moderate | 2 | 4 | 0 | 8 | 11 | 18 | 31 |
|  | High | 0 | 0 | 0 | 0 | 0 | 3 | 8 |

*Table 2B. Association between orthostatic MAP* and educational status

| Orthostatic MAP | Educational status | | | |
| --- | --- | --- | --- | --- |
| Count | | Primary | Secondary | Tertiary |
| 1 min standing | Low | 1 | 7 | 21 |
|  | Moderate | 10 | 30 | 21 |
|  | High | 2 | 6 | 1 |
| 3 min standing | Low | 2 | 5 | 16 |
|  | Moderate | 8 | 33 | 24 |
|  | High | 3 | 5 | 3 |
